# An Overview of the Tympanostomy Tube

**DOI:** 10.7759/cureus.30166

**Published:** 2022-10-11

**Authors:** Rashi R Nagar, Prasad T Deshmukh

**Affiliations:** 1 Otolaryngology - Head and Neck Surgery, Jawaharlal Nehru Medical College, Datta Meghe Institute of Medical Sciences, Wardha, IND

**Keywords:** tube otorrhea, serous otitis media, otitis media with effusion, myringotomy, tympanostomy tube

## Abstract

Otitis media is a disorder of the middle ear, which can occur at any age but is more common among infants and children. The patient usually presents with earaches, impaired hearing, and fever. If antibiotics and decongestants do not suit the patient, a myringotomy can be performed to achieve middle ear aeration. In myringotomy, a slit is created in the tympanic membrane, and fluid is removed with suction. In cases where myringotomy, aspiration, and medical care don't help and the fluid recurs, a tympanostomy tube is inserted to create continuous aeration of the middle ear. A tympanostomy tube is a small tube inserted in the tympanic membrane which helps in the prevention of fluid accumulation in the middle ear. These tubes are temporary and often fall off after the ear heals. Other names for tympanostomy tubes are grommet, myringotomy tube, or pressure equalizing tube. Initially, tympanostomy tubes were made of metal but now fluoroplastic or silicone elastomers are used to make them. The two basic designs of a tympanostomy tube are short-term tube and long-term tube. The choice of a tympanostomy tube depends on factors like age, the period needed for ventilation, socioeconomic status, and the extent of the retracted eardrum. The incidence of occlusion, infection, functional duration, and persistent perforation following extrusion varies between the designs and materials. Every year, many children are affected by recurrent otitis media, which can negatively influence their quality of life and their ability to hear and communicate. With so many children requiring tympanostomy tubes, choosing the appropriate tube is vital to provide optimal treatment and limit complications.

## Introduction and background

One of the most frequently found infections in children is otitis media. Otitis media is the inflammation of the middle ear cleft due to infection or tympanic membrane perforation leading to earache, otorrhea, and impaired hearing. In children, otitis media is a common reason for visiting a doctor, receiving antibiotics, and undergoing surgery [1]. The eustachian tube contributes to the maintenance of normal middle ear function. Any eustachian tube dysfunction can lead to infections [2]. In children, the eustachian tube is smaller in diameter, shorter in length, and at a more acute angle to the nasopharynx. All these reasons predispose children to eustachian tube dysfunction and thus higher infection risk. Eustachian tube blockage due to a mass, allergy, enlarged adenoid, or infection can lead to fluid collection in the middle ear. The fluid in the middle ear can prevent vibration of the tympanic membrane leading to impaired transmission of sound via the middle ear. Inadequate sound transmission due to the presence of fluid can lead to hearing loss. Thus, proper treatment is vital [3]. The treatment regimen includes the use of antibiotics and decongestants. Myringotomy has to be done in severe cases of otitis media with effusion. Myringotomy, also known as a tympanostomy, is a surgical procedure to remove the fluid in the middle ear and re-establish equal air pressure on both sides of the eardrum. The surgeon makes a small slit in the eardrum and drains the fluid in the middle ear cleft [4]. In cases where the incision doesn't drain the fluid completely, or there is a recurrent infection, a small tube is placed through the slit. This small tube is known as a tympanostomy tube, grommet, ventilation tube, or pressure equalizing tube. It drains the fluid that collects and allows air into the middle ear to keep it ventilated. Tympanostomy tubes are placed to maintain tympanic membrane patency so that the middle ear can drain and the negative middle ear pressure is relieved. 

## Review

Objective

This review article aims to overview and discuss the tympanostomy tube (grommet), its indications, composition, designs, and complications.

Tympanostomy tube

In otorhinolaryngological terms, a tympanostomy tube is defined as a tube surgically inserted in the tympanic membrane [5]. It is a tiny structure about two millimetres long with varying dimensions of flanges, inserted in the tympanic membrane to drain persistent fluid and provide ventilation to the middle ear. A radial incision is created on the anteroinferior quadrant of the tympanic membrane to insert the tube. A radial incision/slit is preferred as it holds the tube tightly. The incision in the posterosuperior quadrant can cause injury to important structures of the middle ear cavity. In cases of serous otitis media where it is challenging to drain fluid via an incision in the anteroinferior quadrant, the incision at the anterosuperior quadrant is preferred. These tubes were first used in the late 1800s and were remade using plastic in 1954 by Beverley Armstrong [6]. After that, many advances have been made in the materials used for composition and designs.

Indication

Persistent otitis media with effusion (OME) for more than three months is the major indication for a tympanostomy tube [7]. Serous otitis media (SOM), which does not resolve after three months and is unresponsive to clinical therapy, is also an indication for a tympanostomy tube [8]. Tympanostomy tubes help drain middle ear fluid and provide a ventilatory port to the middle ear cavity [9]. In cases of acute otitis media that is refractory to antibiotic therapy, a tympanostomy tube is indicated. Tympanostomy tubes can also be used for patients who cannot tolerate oral antibiotics. In these cases, tympanostomy drains not only the middle ear fluid but also allows easy access to topical antibiotic drops to the middle ear cavity. Also, in cases of otitis media with effusion where hearing is impaired because of fluid accumulation, a tympanostomy tube is indicated.

Complications of otitis media such as facial nerve palsy, otomastoiditis, and meningitis are additional indications for tympanostomy tubes. Tympanic membrane damage in patients with these issues can be prevented with the help of tympanostomy tubes. Complications of otitis media can damage the tympanic membrane by the formation of retraction pockets, which distort the eardrum, or because of adhesive otitis media, which limits ossicular vibrations and can lead to permanent hearing loss [10]. Tympanostomy tubes should be inserted as soon as possible to ventilate the middle ear area and stop the eardrum from retracting further under negative pressure.

Tube material

The most prevalent materials used to make tympanostomy tubes are plastics and metals [11]. The material should be compatible with the middle ear to work as a tympanostomy tube, as the tube will induce the host patient to have a foreign body reaction [12]. Using a material with a lesser inflammatory response in tube manufacturing increases biocompatibility and thus will be preferred. Many metals have been utilized to make tympanostomy tubes, including stainless steel, gold, and titanium. Fluoroplastic is one of the most often utilized materials in tympanostomy tube manufacture. Polytetrafluoroethylene (PTFE), a material made up of fluorine atoms and carbon atoms, was also used in manufacturing [13]. Silicone is another popular material. It's soft and stretchy and thus can be easily removed [14]. 

Biocompatibility and surface composition of the tube

The capability of implant material to function in vivo without giving rise to negative local or systemic reactions in the body is known as biocompatibility [15]. Biocompatible substances that are tested for safety and effectiveness in tissue and on animals are termed biomaterials. Inert materials can be described as biomaterials that cause no or little host response [16]. The tube must be biocompatible with the middle ear in order to avoid difficulties and maintain the tube's function. As the tubes can cause inflammatory reactions, inert materials like stainless steel or gold are appropriate. Tympanostomy tubes can also be made from other materials, such as fluoroplastic (Teflon, PTFE). They aid in preventing rejection due to their strong heat resistance and chemical inertness. Silicon is a pliable polymer that can withstand a wide range of temperatures since it is made up of silicon and oxygen atoms in an alternate manner. A clinical study among children who received Shepard fluoroplastic tubes in one ear and titanium tubes in another found that the titanium tubes caused more significant granulation tissue and had a higher infection rate [17]. In another study on rats that underwent myringotomy and installation of tympanoplasty tubes, it was discovered that polyethylene tubes caused the most extensive structural changes in the tympanic membrane, followed by stainless steel tubes, and finally fluoroplastic tubes [18]. The purpose of enhancing biocompatibility is to generate less host tissue reaction, leading to a lower occurrence of potential problems, such as infection [19].

Various materials including antibiotics like vancomycin have been coated on tympanostomy tubes to improve biocompatibility and lower the incidence of complications. Tube otorrhea has been reduced by impregnating silicone tubes with silver oxide. Silver oxide smoothens the silicone's surface and make the tube less sticky. Phosphorylcholine (PC) can be applied on Teflon tubes to attract water molecules while repelling other molecules. Antibiotic-coated tubes and albumin-coated tubes are two other options to consider [20].

Tube design

Although there are many different tube designs, they can be classified as short-term and long-term tubes. The structural difference in the long-term and short-term tubes is the presence or absence of an outward flange, with long-term tubes lacking an outer flange. The short-term tubes are designed to stay in the tympanic membrane for eight to 15 months, whereas long-term tubes can stay in the tympanic membrane for 15 to 18 months [21]. The duration of retention time between the tube depends on the outer flange. When the tympanic membrane begins to heal, the tube is pushed posterior-inferiorly as the tympanic membrane's squamous layer keratinizes. Keratin accumulates behind the outer flange of short-term tubes, progressively pushing the tube out. Due to the lack of an outside flange on long-term tubes, keratin fails to build up behind the flange, resulting in extended retention times [22]. The differentiating feature between the two types of tubes is briefly explained in Table 1. 

**Table 1:** Classification of the Tympanostomy Tube

	Tympanostomy Tube Classification	Feature
1.	Short-term tube	Both inward and outward flanges present.
2.	Long-term tube	Inward flange more prominent or outward flange absent

Short-term tube

The most common short-term ventilation tubes are the Donaldson ear tube, Paparella I, Armstrong grommet ventilation tube, Sheehy grommet, Reuter bobbins myringotomy tube, and Shepard myringotomy tube. They have a grommet with two flanges with a short shaft in between them. Young children usually have short-term tubes inserted under a general anaesthetic. They are traditionally inserted via a radial slit in the pars tensa. These tubes can usually stay in the tympanic membrane for 8 to 15 months [23].

Long-term tube

Long-term ventilation tubes withstand extrusion forces and can stay in the tympanic membrane for 15 to 18 months. They can have a more prominent inner flange, for example Paparella II, Per-Lee tube; no outside flange, such as Armstrong bevelled ear tube; or both, for Butterfly tube and Goode T tube. Compared to short-term ventilation tubes, these long-term ventilation tubes have an increased rate of granulation and slow healing perforation. When short-term tubes extrude prematurely in cases of atelectasis or considerable tympanic membrane retraction, long-term canulation is sought and long-term tubes are commonly chosen [24].

Tube removal and retention time

About a year after being implanted, tympanostomy tubes usually extrude. Squamous epithelial migrates and accumulates beneath the tube's outward flange, eventually forcing the inward flange through the tympanic membrane into the external auditory canal. The tubes in the ear can also fall out without notice [25]. The duration a ventilation tube can stay in the tympanic membrane is determined by a number of parameters. The short-term ventilation tubes are designed to last for 8 to 15 months in the ear, whereas long-term tubes are designed to last for more than 15 months to two years. Occasionally, wax buildup causes ear tubes to become jammed. The pipes may become stuck in the eardrum in rare instances. In these circumstances, removing the tube and closing the eardrum are done surgically. The formation of a biofilm is a common cause of tube obstruction and infection [26]. Nowadays, certain dissolvable tympanostomy tubes are developed. Their capacity to dissolve on their own or with specific topical solutions might be beneficial for patients who cannot endure removal, reducing the necessity for an operation theatre. It can be used for patients with unreliable follow-ups [27].

Complications

After the tube insertion, the body can react to the presence of foreign body substances, leading to complications. Complications of tympanostomy tube insertion are prevalent. The most common consequences of tympanostomy tube insertion include tube otorrhea, persistent perforation, tympanic membrane atrophy, tube clogging, myringosclerosis, and tympanosclerosis. However, they are usually insignificant and not severe. As a result, no management is required in the vast majority of these complications. But in some patients, complications of tympanostomy tube insertion can severely impact. Tympanosclerosis can even cause permanent hearing loss [28]. The definition of these complications can be seen in Table 2.

**Table 2:** Definition of common tympanostomy tube complications.

Complication	Definition
Tube otorrhea	Abnormal discharge from ear after placement of tympanostomy tube.
Persistent perforation	Ruptured tympanic membrane which does not heal on its own.
Tube clogging	Blocking of tympanostomy tube by wax, blood, ear discharge from tube otorrhea, or foreign body.
Tympanic membrane atrophy	Decrease in elastic and fibrous fibres of lamina propria, leading to thin and unelastic tympanic membrane.
Myringosclerosis	Pathological condition of tympanic membrane leading to sclerotic plaques and hyaline degeneration.
Tympanosclerosis	Calcification and hardening of tissue of tympanic membrane as well as middle ear.

The most common complication of tympanostomy tube insertion is tube otorrhea. It can arise right after tube placement due to an existing middle ear infection (acute otitis media) or later as a result of subsequent middle ear infections or infectious processes in the ear canal [29]. Otorrhea or ear discharge in these cases can be with a foul odour, fever, and pain. This can impact the quality of life negatively. Treatment for tube otorrhea involves the eradication of bacterial infection using oral antibiotics and antibiotic ear drops. Previously it was thought that water precautions in children with tympanostomy tubes could prevent tympanostomy tube otorrhea. But in new studies, it has been confirmed that water precautions in children with tympanostomy tubes do not prevent tympanostomy tube otorrhea. Also, the most effective treatment for tympanostomy tube otorrhea is antibiotic and corticosteroid ear drops [30].

A small perforation may remain in the tympanic membrane if the tympanostomy tube prematurely falls off or if it has to be removed by surgery. Usually, tympanic membrane perforations heal on their own within a week after the rupture, but in some cases, it may take months to heal [31]. This persistent perforation in the tympanic membrane can lead to infections and even cholesteatoma. Chronic infections and cholesteatoma can lead to the destruction of ear structures, causing hearing loss. Thus, correction of the persistent perforation should be done as soon as possible. The correction of perforation prevents the entry of water into the ear, which causes ear infections and cholesteatoma. This correction of perforation of the tympanic membrane is done surgically, and the process is called tympanoplasty. In tympanoplasty, a graft of temporal fascia or cartilage is used to repair the tympanic membrane [32].

Tympanostomy tube clogging is also commonly seen. The tube becomes briefly ineffective when a tympanostomy tube's lumen becomes clogged. Clogging can happen right after tube installation, particularly in ears with effusion, after an otorrhea episode, or as part of the usual extrusion process. Ototopical drops or peroxide solutions can be used to dissolve some blockages. Clogs in tubes that have been in place for more than six months can be removed under the operating microscope. Mechanical clearance of clogs in extruding tubes is not done [33]. Myringosclerosis is a disease of the tympanic membrane. It is usually caused due to the chronic presence of fluid in the ear. In this condition, some parts of the tympanic membrane appear as yellowish, sclerotic plaques. Within the lamina propria, there is an increase in collagen fibres and hyaline degeneration [34]. Atrophy of the tympanic membrane from tympanostomy tube insertion can also cause impairment in hearing. Another complication of the tympanostomy tube is the early extrusion of the tube. Non-functioning of the tympanostomy tube can also be considered a complication [35].

## Conclusions

Otitis media commonly affects many children and adults worldwide. It can affect the standard of living, especially of children, due to its symptoms and effect on hearing. In cases with ear effusion, a simple surgery myringotomy is performed to drain fluid from the ear and bring back normalcy. If myringotomy alone is not sufficient to resolve the condition, then tympanostomy tubes are used. The main indication for the use of this tube is eustachian tube dysfunction leading to otitis media with effusion or serous otitis media. These tympanostomy tubes provide ventilation, equalize pressure in the middle ear, and prevent further recurrent infections. Different types of tympanostomy tubes are present, with different compositions and designs. Common materials used to construct these tubes are fluoroplastic, silicone, titanium, gold, etc. There are two major designs for tympanostomy tubes: short-term tubes and long-term tubes. Increased biocompatibility of tympanostomy tube materials is necessary to generate fewer post-tympanostomy tube insertion complications. Fluoroplastic and silicon are highly biocompatible materials as compared to metals.

The common prevalence of otitis media worldwide and present knowledge about it suggests that there is a need for further research on tympanostomy tubes so as to increase its efficacy and decrease its complications such as otorrhea and clogging. More inert materials should be identified for their biocompatibility. With the advancement in their composition and designs, biocompatibility can further increase, and complications can be minimized. 
